# On the Maintenance of Life in Limbs Separated from the Body, by the Means of Injections of Blood

**Published:** 1852-10

**Authors:** E. Brown-Sequard

**Affiliations:** Paris


### SELECTIONS.

From the New York Medical Times.

*On the Maintenance of Life in limbs separated from the body, by the means of injections of blood.* By E. Brown-Sequard, M. D., of Paris.

James Phillips Kay has found that blood, injected into limbs of dead animals just after irritability has disappeared, is able to regenerate that vital property. I have gone much farther, and have discovered that, even in limbs having lost their irritability, and having been rigid for several hours, blood is able to regenerate local life.

Moreover, I have found that it is possible to entertain local life for a very long time (more than 41 hours), in one of the limbs of a dead warm-blooded animal. Besides, it is very probable that life may be maintained for months, in limbs separated from the body.

Before relating the last experiment, I must give a short abstract of preceding papers on this subject.*

In many rabbits and Guinea-pigs, I have injected blood in the arteries of limbs, separated from the body and rigid for 15 or 30 minutes, or even one or two hours. I have found that the cadaveric rigidity soon disappeared, and that not only muscular irritability reappeared, but that the motor nerves resumed their faculty of exciting muscles. This last fact is more particularly important, because it is a good proof that the vital property of motor nerves depends on their nutrition, and not on an action of the brain or of the spinal cord.†

In other experiments, I divided the body of a living Guinea-pig into two halves, at the level of the lower border of the kidneys, leaving no communication between the two halves, except by the aorta and the vena cava. I then tied the aorta immediately below the origin of the renew arteries. The muscular irritability gradually diminished and gave way to cadaveric rigidity, in between fifteen and forty minutes, after which the ligature was relaxed, and the circulation re-established in the posterior segment of the body. The rigidity was then observed to disappear little by little, and the muscles and nerves resumed their excitability.

Lastly, in order to ascertain if voluntary movements and sensibility can be restored to limbs that have been in a state of cadaveric rigidity, I have tied the aorta immediately below the origin of the

---

* See Gaz. Med. de Paris, t. 6., 1851, pp. 379 and 421.

† See, for other proofs, my paper entitled, *Researches applied to Physiology and Pathology*, in the Medical Examiner, No. viii., August, 1852, p. 483.

renal artery in many rabbits. After a short time, the sensibility and the voluntary movements had disappeared in the posterior limbs. Irritability lasted about an hour, rigidity supervened in from an hour to an hour and twenty minutes after the ligature of the aorta. The rigidity was permitted to continue for twenty minutes, and then the ligature was relaxed. The circulation, and by it successively, the muscular irritability, the excitability of the motor nerves, the voluntary movements, and the sensibility, were re-established.

It results from this experiment, that not only real life, i. e. mere muscular irritability and excitability of motor nerves, but all the properties and actions of full life, can be restored in limbs that have been in the state called *rigor mortis*, *cadaveric or post-mortem rigidity.*

I have also performed some experiments on two decapitated men. One of them was decapitated for thirteen hours, when I injected blood into the arteries of one of the fore arms. Rigidity was then existing in the muscles of that fore-arm, and in those of the hand. They soon disappeared in these last muscles, and they resumed their irritability. In the other man, the injection was made in the brachial artery, and only fourteen hours after the decapitation. The muscles of the arm and fore-arm, which were perfectly rigid and had lost for some time their irritability, became quickly very irritable, and remained living for many hours.

The blood injected in the first of these men was venous, human, healthy blood (my own blood); in the second, the blood injected was the arterial blood of a dog. It is quite indifferent in these experiments whether we inject blood of the same species, or of another species of animal. I may add that even in cases of transfusion in entire animals, the operation may be successful as well when the blood used belongs to the same species as when it belongs to another species, if the blood has been defibrinated. Bischoff has discovered that it is the fibrin of one species of animal which is a poison for an animal of another species. My experiments have demonstrated the exactitude of Bischoff's opinion.

An important deduction from my researches is that fibrin is not the agent of reparation of muscles. This is completely proved by the fact that defibrinated blood is able to repair muscles. Not only fibrin is not necessary for nutrition of muscles; but when defibrinated blood is injected into the limbs, the blood coming out by the veins, after these vessels and also the capillaries have been completely voided of the remaining blood which was in them, contains a small quantity of fibrin. Therefore, when nutrition takes place in the tissues of a limb, fibrin enters or is produced in the blood circulating in that limb. Is that fibrin originated from the globules, or from the albumen of the injected blood, or from another element of that blood? Does it come from the tissues through which the blood passes? I cannot solve these questions; but I have some good reason for believing that a part, if not the whole, of that new fibrin, comes from the muscles in the capillaries of which the blood passes.

Another interesting fact is that the blood, which was red when ejected, came out nearly black. This change of color takes place as long as putrefaction has not begun in the muscles.*

I have made lately an experiment with the view of ascertaining how long a limb, separated from the body of an animal, may be kept living by the influence of injected blood. I have succeeded in entertaining local life a longer time than 41 hours, in one of the limbs of a rabbit. That animal was a very vigorous, full-grown one. I killed it by hæmorrhage.

Two hours afterwards, the rigidity had begun in most of the muscles of the two posterior limbs, and only a few bundles of muscular fibres had still a slight irritability. A first injection of defibrinated blood was then pushed in the femoral artery of the right posterior limb. Fifteen minutes after the beginning of the injection, local life, *i. e.* irritability, was restored in the limb receiving blood, and cadaveric rigidity had disappeared. The means of testing irritability was the old means used by Glisson, Gorton, and all the experimenters of the last two centuries, I mean a mechanical excitation. I did not use Galvanism, because it exhausts too much muscular irritability, as Autenrieth, Pfaff, and many other observers, have shown long ago. Being aware of this fact, I have always. in my other experiments, used Galvanism as rarely as posssible.

Three hours after the death of the rabbit, irritability was still existing iu the right limb (the injected limb); the left was perfectly rigid and had not the slightest irritability. Half an hour later, rigidity had begun again in the right limb; blood was injected anew and rigidity disappeared, and local life came again. From that moment until 11 o'clock, P. M. (the death had occurred at 6 o'clock A. M., the same day), blood was injected a great many times, and rigidity did not come again, and the vital properties of muscles was maintained. Of course, the left limb during that time remained rigid, and had not the slightest irritability.

From 11 o'clock, P. M., until 6 d'clock, A. M., the succeeding day, an abundant injection of blood was made every 20 or 25 minutes. The irritability was not powerful, but it existed in all the muscles of the limb. There was no rigidity at all. The injections were then made more frequently, once in each quarter of an hour, until 3 o'clock, P. M., at which time I was obliged to stop them during an hour and a half. At half-past four o'clock, I found

---

* See, about this change of color of blood in palsied limbs, my paper in the *Medical Examiner*, No. viii., Aug., 1852, p. 486-97.

the limb rigid; and only a few bundles of muscular fibres were still irritable. A very abundant injection was then practiced, and rigidity soon disappeared, giving way to irritability. From this time to 11 o'clock, P. M., a great many injections were made, and irritability was maintained. I was then obliged to give up the experiment. At that moment irritability was strong in all the muscles of the injected limb, except some parts of their pelvic extremities that had n't received a sufficient quantity of blood. The next morning, that limb was in full and energetic rigidity. The other limb had already lost its rigidity, and it had an evident smell of putrefaction. The third day after the death of the animal, rigidity was strong in the injected limb, while the other was in an advanced putrefaction.

If we compare these two limbs, we find that the injected one had a strong irritability at the end of 41 hours after the death of the animal; that it was in full rigidity at the 48th hour; that its rigidity gave way to putrefaction only at the 94th hour. The other limb was in full rigidity at the 5th hour after the death of the animal; its rigidity gave way to putrefaction at the 48th hour; and it was in complete putrefaction at the 70th hour.

From this experiment it results that local life may be maintained more than 41 hours in a limb of an animal, by mere injections of blood. No doubt that it would have remained longer if I had continued the injections. How long could life have been maintained? I cannot answer this question positively, but I believe that life could remain for months in limbs separated from the body of an animal. I am led to this supposition by the preceding experiments, and by the results of many other experiments which prove that the nervous system is not necessary to nutrition.*

In the experiment above related, I have used venous defibrinated human blood, and arterial blood of three rabbits. It is nearly indifferent whether we inject venous or arterial blood, but it is absolutely necessary to inject red blood, *i. e.* oxygenated blood. Oxygen is necessary, either because it prevents the alteration of the globules, or because it acts directly on muscles, as Gustavus Liebig has found it does on their external surface when exposed to air. I believe that oxygen is necessary for these two reasons.

Before finishing, I will point out an evident consequence of my experiments on the revivification of muscles. E. Bruecke has given not long ago a new theory of the cadaveric rigidity. He believes that the fluid existing between the muscular fibres, in the cellular tissue, is liquid fibrin, and that the rigidity of muscles is simply the result of the coagulation of that fibrin. This theory has been admitted by many physiologists, and especially

* See my papers in the *Medical Examiner*, Philadelphia, No. 5, May, 1852, p. 21. No. 8, Aug., 1852, p. 486-97.

by the celebrated Du Bois-Reymond. My experiments prove that this theory is false; for if there was fibrin coagulated around the fibres of the muscles, certainly that fibrin should not be rendered liquid again by an action of blood injected in the muscular blood vessels.

